# Association of Scene Time Interval and Field Arrival to Epinephrine Administration Time with Outcomes in Cardiac Arrest

**DOI:** 10.3390/jcm14186645

**Published:** 2025-09-20

**Authors:** Yohei Okada, Ki Jeong Hong, Marcus Eng Hock Ong, Sang Do Shin, Kyoung Jun Song, Jeong Ho Park, Young Sun Ro, Nur Shahidah, Shir Lynn Lim, Fahad Javaid Siddiqui

**Affiliations:** 1 Department of Preventive Services, Graduate School of Medicine, Kyoto University, Kyoto 606-8501, Japan; yohei_ok@duke-nus.edu.sg; 2 Pre-Hospital and Emergency Research Centre, Duke-NUS Medical School, Singapore 169857, Singapore; marcus.ong@duke-nus.edu.sg (M.E.H.O.); nur.shahidah@duke-nus.edu.sg (N.S.); shir.lynn.lim@nus.edu.sg (S.L.L.); fahad.siddiqui@duke-nus.edu.sg (F.J.S.); 3 Department of Emergency Medicine, Seoul National University Hospital, Seoul 03080, Republic of Korea; shinsangdo@gmail.com (S.D.S.); timthe@gmail.com (J.H.P.); ro.youngsun@gmail.com (Y.S.R.); 4 Department of Emergency Medicine, Seoul National University, Seoul 08826, Republic of Korea; 5 Laboratory of Emergency Medical Services, Seoul National University Hospital Biomedical Research Institute, Seoul 03080, Republic of Korea; skciva@gmail.com; 6 Disaster Medicine Research Center, Seoul National University Medical Research Center, Seoul 03080, Republic of Korea; 7 Department of Emergency Medicine, Singapore General Hospital, Singapore 169608, Singapore; 8 Department of Emergency Medicine, Seoul National University Boramae Medical Center, Seoul 03080, Republic of Korea; 9 Department of Emergency Medicine, Seoul National University College of Medicine, Seoul 03080, Republic of Korea; 10 Department of Cardiology, National University Heart Centre, Singapore 119074, Singapore; 11 Yong Loo Lin School of Medicine, National University of Singapore, Singapore 117597, Singapore

**Keywords:** out of hospital cardiac arrest, epinephrine, scene time interval, survival, good neurologic recovery

## Abstract

**Background/Objectives:** The association of scene time interval (STI) and field arrival to epinephrine administration time (FET) with outcomes in out-of-hospital cardiac arrest (OHCA) is unknown. The goal of this investigation is to assess the association of STI and FET with outcomes in OHCA. **Methods**: All adult OHCA cases with prehospital epinephrine administration in South Korea and Singapore were included. STI was divided into short and long stay based on the median value of each country. FET was categorized into early (<10 min) and late groups. We performed multivariable logistic regression for survival to discharge and good neurological recovery. Cases were grouped into short stay early epinephrine (SS-EE), short stay late epinephrine (SS-LE), long stay early epinephrine (LS-EE), and long stay late epinephrine (LS-LE) (reference). Interaction analysis with STI and FET for outcomes was conducted. **Results:** A total of 18,867 cases from South Korea and 4184 cases from Singapore were included. Adjusted odds ratio (AOR) for survival to discharge was 2.14 (95% CI: 1.18–2.25) in SS-EE, 1.15 (0.94–1.40) in SS-LE, and 1.82 (1.45–2.28) in LS-EE compared to LS-LE in South Korea with similar results for Singapore. SS-EE and LS-EE were also associated with good neurologic recovery. Interaction analysis showed that early epinephrine injection in short stay and long stay was associated with better outcomes. But short STI was not associated with better outcomes in early and late epinephrine groups. **Conclusions:** Early epinephrine administration was associated with higher survival to discharge irrespective of the scene time interval.

## 1. Introduction

Out-of-hospital cardiac arrest (OHCA) is a major public health problem with high mortality [1]. Intravenous epinephrine administration has been used for OHCA patients during prehospital resuscitation by paramedics in the United States and other developed countries. According to the latest European Resuscitation Council guidelines, epinephrine should be administered as soon as possible for patients with non-shockable rhythms, and after the third defibrillation attempt for shockable rhythms, while ensuring that chest compressions and other resuscitation measures are not interrupted [2]. However, evidence for the optimal time of prehospital epinephrine administration is still being debated [3]. Field arrival to the first epinephrine dose administration time (FET) in prehospital settings is associated with better clinical outcomes, such as survival to discharge when FET is less than 10 min [4]. Training and expertise in obtaining intravascular access, as well as a high-performance team-based cardiopulmonary resuscitation (CPR) are required for the rapid administration of epinephrine in the prehospital setting.

Scene time interval (STI) defined as time from scene arrival to departure is an important quality indicator of prehospital resuscitation. Optimal STI for OHCA is also unknown. STI varies across different Emergency Medical Services (EMS) systems globally, depending upon the experience or local evidence of the individual systems [5]. In Asian countries like South Korea, prehospital use of medications other than epinephrine is prohibited and all OHCA cases are transported even though prehospital return of spontaneous circulation (ROSC) has not been achieved. The same practice is followed in Singapore. In the developed countries with prehospital advanced life support (ALS), requests for intra-arrest transport are recently increasing due to the application of extracorporeal membrane oxygenation (ECMO) CPR application.

To improve the prehospital resuscitation quality and clinical outcomes for OHCA patients, comprehensive quality improvement of time-sensitive prehospital interventions is required. Early prehospital epinephrine administration and optimal STI are recommended for EMS systems but the optimal combination of both FET and STI for better clinical outcome has not been investigated [6,7]. Application of early FET or short STI in the prehospital setting could require different EMS protocols, education, and emergency medical resources.

The aim of this study is to assess the association of STI and FET with OHCA patients’ clinical outcomes in South Korea and Singapore using retrospective nationwide OHCA registries. We hypothesized that early epinephrine administration and shorter STI would be associated with better clinical outcomes in OHCA patients.

## 2. Materials and Methods

### 2.1. Study Design

This investigation was a retrospective observational study using nationwide OHCA databases including the Korean Out-of-Hospital Cardiac Arrest Registry (KOHCAR) for South Korea and the Pan-Asian Resuscitation Outcome Study (PAROS) for Singapore [8,9]. The dataset access and study protocol using KOHCAR were approved by the institutional review board in South Korea with a waiver of informed consent (IRB no. H-1103-153-357, approval date: 8 June 2011). KOHCAR data used in this study were anonymized observational data collected by a retrospective medical record review. This data collection and analysis was minimal risk research involving a medical record review and retrospective observational methods. This observational data collection did not result in changes in prehospital emergency procedure or medications. The data access using the Singapore PAROS dataset was exempted by the Centralised Institutional Review Board (CIRB ref: 2018/2937, approval date: 16 October 2018) due to the use of de-identified data.

### 2.2. Study Setting

This study included all adult EMS-treated OHCA cases in South Korea and Singapore. The EMS systems of South Korea and Singapore were similar with respect to the operational model, i.e., publicly funded and operated by the fire department. Both EMS systems employed relatively limited prehospital ALS with recent implementation of prehospital epinephrine injection and intra-arrest transport protocols.

The EMS system of South Korea is a nation-wide, single-tiered public service model operated by the National Fire Agency. The usual level of prehospital emergency care is emergency medical technician (EMT) intermediate. For OHCA cases, a two-tiered response is adopted with fire engines or ambulances. Advanced airway management including supraglottic airway or endotracheal intubation, intravenous access for hydration and epinephrine, automatic electrical defibrillator and mechanical chest compression are performed for prehospital resuscitation. Prehospital use of epinephrine by EMTs was permitted for the first time in 2019 during the nationwide pilot project aiming to expand the scope of prehospital care [8]. During the project, certified EMS providers could administer epinephrine during prehospital resuscitation under direct medical oversight. After the pilot project, intravenous epinephrine injection by EMTs during the prehospital period became standard of care. Now, all EMS-treated OHCA patients should be transported to the nearest level 1 or level 2 emergency center with ongoing CPR regardless of prehospital ROSC acquisition except in cases of termination of resuscitation.

In Singapore, a nation-wide EMS system is provided by the Singapore Civil Defence Force (SCDF), and activated by a universal, centralized ‘995’ dispatch center. Paramedics are EMT-intermediate trained and able to provide defibrillation, perform advanced airway procedures, and administer intravenous medications such as epinephrine [9]. The administration of intravenous epinephrine for OHCA was approved by Singapore’s Ministry of Health (MOH) and SCDF’s Medical Advisory Committee on 15 October 2003. Intravenous epinephrine is administered after CPR initiation and initial defibrillation according to advanced cardiac life support guidelines [10]. All EMS-treated OHCA patients are conveyed to the nearest tertiary hospital except for cases pronounced dead at the scene following the termination of the resuscitation protocol.

### 2.3. Data Source

This study utilized two separate data sources: KOHCAR for OHCA cases which occurred in South Korea and PAROS for cases which occurred in Singapore. Two analysis datasets were generated and analyzed independently by researchers designated by the IRB of each dataset. KOHCAR is a nationwide OHCA registry covering all OHCA cases transported by EMS in South Korea from 2006 [11]. The KOHCAR collected prehospital clinical data from the dispatch registry, EMS run sheet, EMS cardiac arrest in-depth registry including the Utstein variables and merged with in-hospital clinical information from hospital medical records. Prehospital information like EMS time profiles, Utstein variables and epinephrine administration was recorded during prehospital care by EMTs of the National Fire Agency and registered to KOHCAR. In-hospital resuscitation-related information and outcomes were collected by trained medical record reviewers of the Korea Disease Control and Prevention Agency. Quality management committees of KOHCAR consisting of emergency physicians and statistical experts ensured a quality improvement process.

PAROS is an Asia-Pacific clinical research network that was established in 2010 [12]. Data variables collected follow the Utstein definitions for OHCA which include pre-hospital data fields and other time-sensitive OHCA data elements. Data collected are entered into a secured online electronic data capture system developed in collaboration with the United States Center for Disease Control and the Cardiac Arrest Registry to Enhance Survival. The network is managed by a PAROS Data Administrator and Network Secretary based in Singapore. The PAROS Data Administrator manages the online data entry system and performs additional data quality audits. In Singapore, OHCA data from EMS and eight participating tertiary hospitals are collected prospectively by research coordinators following data quality checks and verification. Linkage between EMS and hospital data is performed by the Unit for Prehospital Emergency Care, under the Ministry of Health, Singapore, the custodian of Singapore’s national OHCA registry [13]. The de-identified data is then added to the PAROS network registry for international collaboration. Only Singapore data was used for this study.

### 2.4. Selection of Participants

The study population included all EMS-treated adult OHCA patients (≥18 years old) who received prehospital intravenous epinephrine in South Korea and Singapore. The study period for South Korea was from July 2019 to December 2022, and for Singapore was from January 2018 to December 2020. We excluded pediatric OHCA, cases with no or unknown receipt of pre-hospital epinephrine, or the cases where EMS call to epinephrine injection time, FET, or STI is <0 min or >60 min or unknown, as well as cases with unknown survival outcomes at hospital discharge.

### 2.5. Variables and Measurements

The main exposures considered in this study were STI and FET. Cases were divided into a short stay group and a long stay group based on median STI values of each country. Using FET, cases were divided into an early epinephrine group and a late epinephrine group; this was based on two different cut-offs: using a threshold of 10 min and median FET of each country [4]. We applied early FET classification by <10 min for the main analysis. We also performed an exploratory analysis using FET categorized by the median value in each country. We combined both STI and FET categories and reclassified the cases into four groups: short stay, early epinephrine (SS-EE); short stay, late epinephrine (SS-LE); long stay, early epinephrine (LS-EE); and long stay, late epinephrine (LS-LE).

We collected demographic variables including age, gender, and the year of cardiac arrest occurrence. We also collected prehospital resuscitation variables such as location of cardiac arrest, witness information, bystander CPR, primary cardiac rhythm at the scene, EMS defibrillation status, and prehospital advanced airway management. Response time, STI, transport time, and FET were recorded in units of minutes. The number of prehospital epinephrine administration was collected and categorized as 1, 2, and ≥3. In-hospital resuscitation-related variables included percutaneous coronary intervention, targeted temperature measurement, and ECMO. For clinical outcomes, any ROSC, survival to discharge, and Cerebral Performance Category (CPC) 1–5 were analyzed.

### 2.6. Outcome

The primary outcome was survival to hospital discharge. The secondary outcome was good neurological recovery defined as CPC 1 (good cerebral performance; ability to work) or 2 (moderate cerebral disability; ability to perform daily activities independently).

### 2.7. Statistical Analysis

We reported categorical variables as number and percentage. Categorical variables were compared by the chi-squared test. We calculated age, EMS time variables including STI or FET by median value and interquartile range (IQR). Continuous variables were compared by Wilcoxon rank-sum test. *p*-values < 0.05 were considered statistically significant. We performed multivariable logistic regression to assess the association of STI and FET with clinical outcomes. Adjusted odds ratios (AORs) with 95% confidence intervals (CIs) were analyzed. Confounders adjusted in the multivariable logistic regression were age by 10 years, gender, witnessed status, bystander CPR, initial shockable rhythm, prehospital advanced airway, prehospital defibrillation, number of prehospital epinephrine injections, response time, transport time, study period by year. To assess the effect of STI category and FET category on clinical outcomes, an interaction analysis was conducted. The interaction terms between short versus long stay and early versus late epinephrine (STI category × FET category) was added to the previous regression model. Statistical analysis for KOHCAR data was performed using SAS version 9.4 (SAS Institute, Inc., Cary, NC, USA) and analysis for Singapore PAROS was conducted by R version 1.1.456 (R Project for Statistical Computing).

## 3. Results

**Figure 1:**
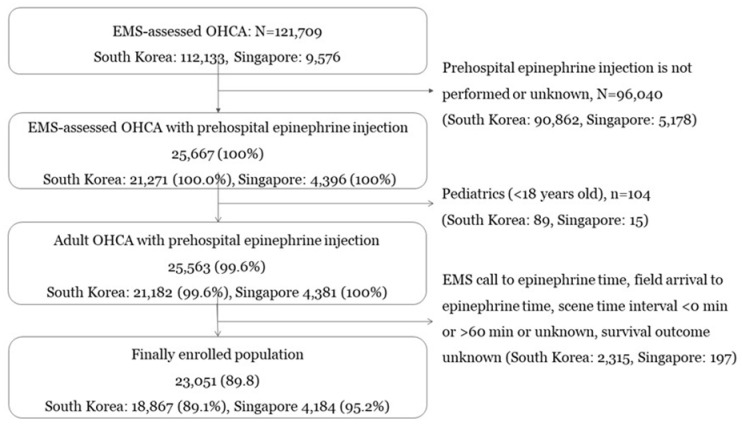
Study population inclusion criteria. This figure illustrates the patient selection flow. A large proportion of registered cases (90,862 of 112,133) are excluded because prehospital epinephrine was not administered. Also, cases with negative time values or implausible values (e.g., greater than 60 min) are excluded from the analysis. The characteristics of the patients excluded from the analysis are indicated in Table A1.

A total of 21,271 EMS-treated OHCA cases from South Korea and 4396 cases from Singapore underwent prehospital epinephrine injection during the retrospective study period. Based on the inclusion/exclusion criteria, 18,867 cases from South Korea and 4184 cases from Singapore were finally included (Figure 1). OHCA patient, OHCA event, and EMS response characteristics were similar in the two countries (Table 1). Proportion of cases with any ROSC was 37.5% and 31.4% in South Korea and Singapore. Survival to discharge was 5.9% and 2.5%, and good neurologic recovery was 2.8% and 1.3% in South Korea and Singapore, respectively. Among the excluded OHCA patients in Singapore aged ≥18 years, the proportion with good neurological recovery was 6.3% (322/5084) and the proportion who survived to discharge was 7.9% (403/5084). Among the excluded OHCA patients in Korea aged ≥ 18 years, the proportion with good neurological recovery was 5.4% (4769/89,081), and the proportion who survived to discharge was 8.1% (7180/89,081).

Median STI (IQR) was 18 min in South Korea and 24 min in Singapore. The short stay group of South Korea was defined as patients with STI of less than 18 min and the short stay group in Singapore was defined as patients with STI of less than 24 min. Median FET (IQR) was 11 min and 14 min in South Korea and Singapore. The proportion/fraction of OHCA patients receiving prehospital epinephrine injections ≥ 3 times was 59.0% and 45.7% in South Korea and Singapore, respectively. In South Korea, highest survival to discharge was observed in the SS-EE (7.7%) and LS-EE (6.9%) groups (Table 2). The highest survival in Singapore was in the SS-EE (4.3%) and LS-EE (7.3%) groups. Proportions of patients with good neurological recovery in the SS-EE and LS-EE groups were 3.8% and 3.5% in South Korea and 1.6% and 5.3% in Singapore, respectively.

**Figure 2:**
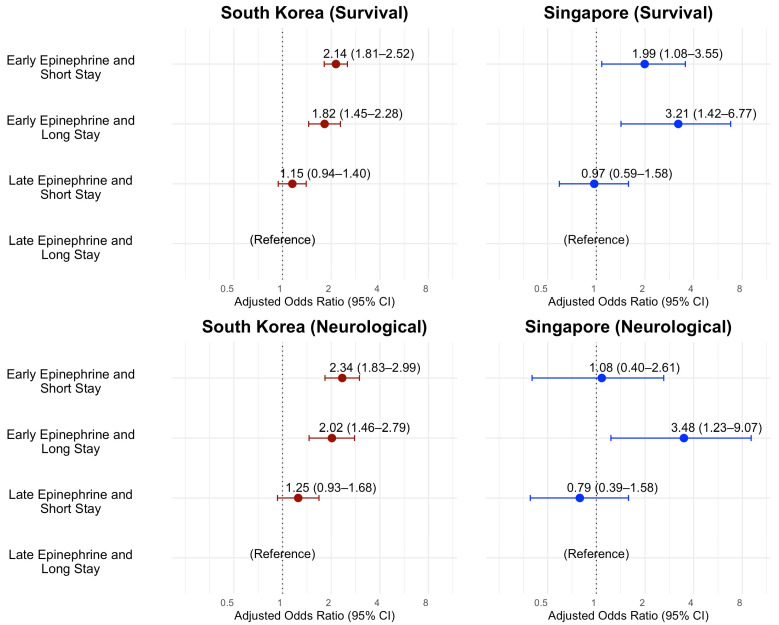
Forest plots for association of scene time interval and field time interval with clinical outcome by multivariable logistic regression. Adjusted odds ratios with 95% confidence interval (CI) are shown. X-Axis: adjusted odds ratio (95% confidence interval), log-scale.

In the multivariable logistic regression model for survival to discharge in South Korea, AORs and (95% CIs) of SS-EE were 2.15 (1.81, 2.52) and AORs of LS-EE were 1.82 (1.45, 2.28) compared to LS-LE as reference (Table 3, Figure 2). The SS-LE group was not significantly associated with survival to discharge. AORs of survival to discharge in Singapore were also higher in the SS-EE and LS-EE group by 1.99 (1.08, 3.55) and 3.21 (1.42, 6.77). SS-LE was not also associated with better survival to discharge in Singapore. In multivariable logistic regression for good neurologic recovery, the SS-EE and LS-EE group of South Korea showed higher AORs and the SS-LE group was not significant. LS-EE was associated with better good neurologic recovery and AORs of SS-EE were not significant by 1.08 (0.40, 2.61) in Singapore. The SS-LE group of Singapore was not significant.

**Figure 3:**
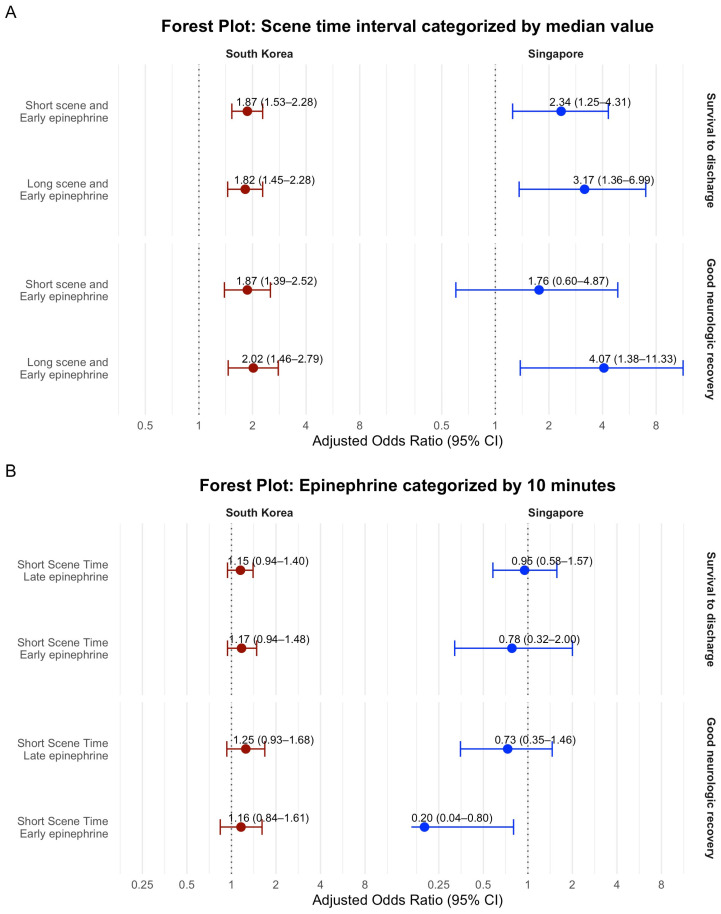
Forest plots for interaction analysis for (**A**) scene time interval categorized by median value and (**B**) epinephrine administration by 10 min with clinical outcome. Adjusted for age by 10 years, gender, witness, bystander CPR, initial shockable rhythm, prehospital advanced airway, prehospital defibrillation, number of prehospital epinephrine injections, response time, transport time, study period by year.

In the interaction analysis of both countries for survival to discharge, the early epinephrine group showed higher AORs for survival to discharge in SS and LS groups (Table 4, Figure 3). And SS was not associated with higher survival to discharge in early or late epinephrine group in the interaction analysis of both countries. In the South Korea data, interaction for good neurologic recovery, early epinephrine administration was associated with good neurologic recovery in SS and LS groups. In Singapore, in the interaction analysis of neurologic outcome, early epinephrine injection in the LS group was associated with good neurologic recovery. The short STI group was associated with lower good neurologic recovery in the early epinephrine group. The results of multivariable logistic regression and interaction analysis using cut-off value of FET by median value of each country are described in the Appendix A.

## 4. Discussion

This investigation assessed the association of STI and FET with clinical outcomes in OHCA patients from South Korea and Singapore. Compared to the long stay and late epinephrine administration group, early epinephrine administration was associated with higher survival to discharge regardless of STI in both EMS systems. The short-stay and late-epinephrine group was not associated with survival to discharge. In the interaction analysis, early epinephrine had positive association for better survival to discharge in short or long STI in both EMS systems. However, short scene stay was not associated with higher survival to discharge in either the early or late epinephrine administration groups. This is one of the few studies evaluating the association between two important prehospital time quality indicators and clinical outcomes of OHCA cases who received prehospital epinephrine.

Prehospital epinephrine administration was associated with better clinical outcomes in previous studies when epinephrine was given soon after scene arrival [2,3,4,6,7,14,15,16]. Delayed epinephrine administration also had been shown to worsen cerebral perfusion during cardiac arrest [17]. If EMS protocols allow for prehospital epinephrine use, early epinephrine administration would be recommended to increase the quality of prehospital resuscitation.

Cut-off values for optimal time for prehospital epinephrine injections vary across EMS protocols and prehospital resuscitation-related environments of each community. In this study, we selected 10 min as the cut-off for FET based on previous literature [4]. However, optimal FET could be determined based on EMS resources, manpower, tiering system, response time, and scope of prehospital emergency procedures of each community. Therefore, we also performed an additional analysis using the median FETs as cutoff values extracted from corresponding databases (Table A2, Table A3 and Table A4).

In the multivariable logistic regression using median FETs as cut-off, SS-EE and LS-EE were also associated with higher survival to discharge, and SS-LE was not associated with survival in both countries. The dose, interval, cumulative number of epinephrine injections, and method of epinephrine administration like intraosseous or intravenous access could affect the result [16,18,19,20]. In this study, prehospital epinephrine was only given by intravenous access, and the number of prehospital epinephrine injections was adjusted as a confounder for logistic regression.

The optimal STI and impact of STI on clinical outcomes in OHCA is still unclear [5,21,22]. If scope of practice in the prehospital resuscitation includes the implementation of prehospital epinephrine administration, it could lengthen the time of scene stay. If EMS protocol of the community does not include prehospital ALS, the OHCA victim should be transported to the hospital regardless of prehospital ROSC in developing EMS systems. Prolonged STI was associated with worsened OHCA outcomes in previous investigations from Asian countries [5]. Optimal STI has also been investigated considering the optimal delivery of post resuscitation care and ECMO CPR in advanced EMS settings [23,24,25]. In this study, SS was not associated with better clinical outcomes compared to the positive association of the early epinephrine group with better outcomes.

This investigation assessed the combined association of both FET and STI on clinical outcomes of OHCA. We found that early epinephrine administration is more important to increase survival to discharge in OHCA rather than reducing length of stay at the scene in this study. Previous studies selected either FET or STI as the main exposure and the other was used as a confounder. However, EMS protocols and the prehospital resuscitation setting affect both STI and FET. And both time profiles could affect the process of prehospital resuscitation and clinical outcome together. In the interaction analysis for this study, early epinephrine showed positive association with survival to discharge, but short stay was not significant. Therefore, EMS quality improvement should focus on how to reduce scene arrival to first epinephrine rather than STI.

### Limitations

This study has several limitations. First, although this study utilized data from two countries, Singapore and Korea, their EMS systems share key operational similarities: both are fire-based, nationwide, single-tier public EMS systems in which advanced life support (ALS) is provided by paramedics. Therefore, the risk of systemic bias arising from fundamental EMS structural differences is considered limited; nevertheless, minor differences in operational protocols, training emphasis, and resource availability may still influence the results. Second, this study was a retrospective observational study and was limited to the variables available in the databases, with some measurement variability. EMS time profiles were recorded by EMS providers during prehospital resuscitation, and there is a possibility of inaccuracies in these time measurements. However, these inaccuracies are most likely random, and the main effect would be slightly wider confidence intervals. Information about prehospital performance indicators, such as the success rate of intravenous access, advanced airway management, and quality of chest compression, was not available and therefore could not be adjusted for. Post-OHCA survival also depends on advanced interventions received after admission to the hospital, but we did not have data to adjust for this factor. Third, the study periods differed between the two countries (2019–2022 for Korea and 2018–2020 for Singapore). Although limiting the analysis to common years could reduce the potential impact of organizational changes and external factors such as the COVID-19 pandemic, the small sample size in Singapore would have resulted in very few outcome events in each year, making meaningful year-by-year analyses unfeasible. Finally, the cut-off values are all arbitrary and hence these results will be generalizable for the EMS systems similar to the ones in this analysis. To assess the association of time profiles, scene time, and clinical outcomes more accurately, perhaps a randomized prospective trial is required.

## 5. Conclusions

Early epinephrine administration was associated with higher survival to discharge irrespective of the scene time interval. In contrast, shorter STI was not associated with higher survival to discharge in either the early or late epinephrine administration groups. Caution must be exercised when these results are applied to differently organized and operated EMS systems. Further research with greater methodological rigor is required.

## Figures and Tables

**Table 1:** Demographic finding and clinical information.

Variables		South Korea		Singapore	
		N = 18,867		N = 4184	
		N	%	N	%
Age	median (IQR)	71.4 (58.7, 81.3)		71.0 (59.0, 82.0)	
Gender	male	12,679	67.2	2800	66.9
	female	6188	32.8	1384	33.1
Year	2018	-	-	1417	33.9
	2019	742	3.93	1430	34.2
	2020	6036	31.99	1337	32.0
	2021	5976	31.67	-	-
	2022	6113	32.4	-	-
Witnessed arrest	yes	8335	44.2	2152	51.4
	no	10,532	55.8	2032	48.6
Location of arrest	private	11,754	62.3	3483	83.2
	public	7113	37.7	701	16.8
Bystander CPR	yes	12,110	64.2	2612	62.4
	no	6757	35.8	1572	37.6
Initial shockable rhythm	yes	2724	14.4	719	17.2
	no	16,143	85.6		
Prehospital defibrillation	yes	4309	22.8	1104	26.4
	no	14,558	77.2	3080	73.6
Advanced airway management	yes	18,684	99.0	4075	97.4
	no	183	1.0	109	2.6
PCI	yes	1040	5.5	109	2.6
	no	17,827	94.5	4074	97.4
TTM	yes	921	4.9	234	5.6
	no	17,946	95.1	3949	94.4
ECMO	yes	277	1.5	12	0.3
	no	18,590	98.5	4171	99.7
Any ROSC	yes	7066	37.5	1315	31.4
	no	11,801	62.6	2868	68.6
Survival to discharge	yes	1107	5.9	105	2.5
	no	17,760	94.1	4079	97.5
Good neurologic recovery	CPC 1, 2	526	2.8	53	1.3
	CPC 3, 4, 5	18,341	97.2	4131	98.7

**Table 2:** Prehospital resuscitation and EMS time profiles.

			South Korea (N = 18,867)					Singapore (N = 4184)				
			No		Yes		*p*-Value	No		Yes		*p*-Value
			N	%	N	%		N	%	N	%	
Survival to discharge			17,760	94.1	1107	5.9		4079	97.5	105	2.5	
number of prehospital epinephrine injection		1	2864	87.5	409	12.5	<0.01	933	95.0	49	5.0	<0.01
		2	4139	92.6	330	7.4		1261	97.9	27	0.1	
		≥3	10,757	96.7	368	3.3		1885	98.5	29	1.0	
response time (min)		median, IQR	9	7–12	8	6–10	<0.01	9	7–11	8	7.10	0.15
scene time interval (STI) (min)		median, IQR	18	15–23	17	14–21	<0.01	24	21–28	23	19.27	0.04
transport time (min)		median, IQR	7	4–11	7	5–12	0.14	6	4–9	6	4.8	0.6
field arrival to epinephrine time (FET) (min)		median, IQR	11	8–14	10	7–13	<0.01	14	11–20	11	9.16	<0.01
STI categorized by median value and FET categorized by 10 min	short stay and early epinephrine		4772	92.3	399	7.7	<0.01	466	95.7	21	4.3	<0.01
	short stay and late epinephrine		3437	94.5	201	5.5		1399	97.6	34	2.4	
	long stay and early epinephrine		1851	93.1	137	6.9		140	92.7	11	7.3	
	long stay and late epinephrine		7700	95.4	370	4.6		2074	98.2	39	1.8	
Good neurologic recovery			18,341	97.2	526	2.8		4131	98.7	53	1.3	
number of prehospital epinephrine injection		1	3045	93.0	228	7.0	<0.01	954	97.1	28	2.9	<0.01
		2	4323	96.7	146	3.3		1275	99.0	13	1.0	
		≥3	10,973	98.6	152	1.4		1902	99.4	12	0.6	
response time (min)		median, IQR	9	6–12	8	6–10	<0.01	9	7–11	8	7.10	0.14
scene time interval (min)		median, IQR	18	15–23	17	13–20	<0.01	24	21–28	24	21.29	0.8
transport time (min)		median, IQR	7	4–11	7	5–13	0.02	6	4–9	6	5.8	0.9
field arrival to epinephrine time (min)		median, IQR	11	8–14	9	7–12	<0.01	14	11–19	11	9.16	<0.01
STI categorized by median value and FET categorized by 10 min	short stay and early epinephrine		4973	96.2	198	3.8	<0.01	479	98.4	8	1.6	<0.01
	short stay and late epinephrine		3545	97.4	93	2.6		1417	98.9	16	1.1	
	long stay and early epinephrine		1919	96.5	69	3.5		143	94.7	8	5.3	
	long stay and late epinephrine		7904	97.9	166	2.1		2092	99.0	21	1.0	

**Table 3:** Multivariable logistic regression of scene time interval and field arrival to epinephrine time for clinical outcome in OHCA.

		Total	Outcome		Unadjusted			Adjusted *		
			N	%	OR	95% CI		AOR	95% CI	
South Korea										
Survival to discharge		18,867	1107	5.9						
Median STI and FET by 10 min	short stay early epinephrine	5171	399	7.7	1.74	1.50	2.01	2.14	1.81	2.52
	short stay late epinephrine	3638	201	5.5	1.22	1.02	1.45	1.15	0.94	1.40
	long stay early epinephrine	1988	137	6.9	1.54	1.26	1.89	1.82	1.45	2.28
	long stay late epinephrine	8070	370	4.6	reference			reference		
Good neurologic recovery		18,867	526	2.8						
Median STI and FET by 10 min	short stay early epinephrine	5171	198	3.8	1.90	1.54	2.34	2.34	1.83	2.99
	short stay late epinephrine	3638	93	2.6	1.25	0.97	1.62	1.25	0.93	1.68
	long stay early epinephrine	1988	69	3.5	1.71	1.29	2.28	2.02	1.46	2.79
	long stay late epinephrine	8070	166	2.1	reference			reference		
Singapore										
Survival to discharge		4184	105	2.5						
Median STI and FET by 10 min	short stay early epinephrine	487	21	4.3	2.4	1.37	4.07	1.99	1.08	3.55
	short stay late epinephrine	1433	34	2.4	1.29	0.81	2.06	0.97	0.59	1.58
	long stay early epinephrine	151	11	7.3	4.18	2.00	8.07	3.21	1.42	6.77
	long stay late epinephrine	2113	39	1.8	reference			reference		
Good neurologic recovery		4184	53	1.3						
Median STI and FET by 10 min	short stay early epinephrine	487	8	1.6	1.66	0.69	3.64	1.08	0.40	2.61
	short stay late epinephrine	1433	16	1.1	1.12	0.58	2.15	0.79	0.39	1.58
	long stay early epinephrine	151	8	5.3	5.57	2.28	12.34	3.48	1.23	9.07
	long stay late epinephrine	2113	21	1.0	reference			reference		

* Adjusted for age by 10 years, gender, witness, bystander CPR, initial shockable rhythm, prehospital advanced airway, prehospital defibrillation, number of prehospital epinephrine injections, response time, transport time, study period by year. Abbreviation: STI: scene time interval, FET: field arrival to epinephrine time.

**Table 4:** Interaction analysis for scene time interval and epinephrine administration time with clinical outcome.

Country	Outcome		Scene Time Interval Categorized by Median Value					
			Short Scene Time Interval			Long Scene Time Interval		
			AOR	95% CI		AOR	95% CI	
South Korea	Survival to discharge	early epinephrine by 10 min	1.87	1.53	2.28	1.82	1.45	2.28
		late epinephrine by 10 min	reference			reference		
	Good neurologic recovery	early epinephrine by 10 min	1.87	1.39	2.52	2.02	1.46	2.79
		late epinephrine by 10 min	reference			reference		
Singapore	Survival to discharge	early epinephrine by 10 min	2.34	1.25	4.31	3.17	1.36	6.99
		late epinephrine by 10 min	reference			reference		
	Good neurologic recovery	early epinephrine by 10 min	1.76	0.60	4.87	4.07	1.38	11.33
		late epinephrine by 10 min	reference			reference		
			Epinephrine categorized by 10 min					
			Early epinephrine			Late epinephrine		
South Korea	Survival to discharge	short scene time interval	1.17	0.94	1.48	1.15	0.94	1.40
		long scene time interval	reference			reference		
	Good neurologic recovery	short scene time interval	1.16	0.84	1.61	1.25	0.93	1.68
		long scene time interval	reference			reference		
Singapore	Survival to discharge	short scene time interval	0.78	0.32	2.00	0.95	0.58	1.57
		long scene time interval	reference			reference		
	Good neurologic recovery	short scene time interval	0.20	0.04	0.80	0.73	0.35	1.46
		long scene time interval	reference			reference		

Adjusted for age by 10 years, gender, witness, bystander CPR, initial shockable rhythm, prehospital advanced airway, prehospital defibrillation, number of prehospital epinephrine injections, response time, transport time, study period by year.

## Data Availability

The data presented in this study are available on request from the corresponding author. The data used in the study are not publicly available due to privacy and data curation from different institutions from South Korea and Singapore. For public use, approval by PAROS CRN in Singapore and Korea Disease Control and Prevention Agency could be required.

## Appendix A

**Table A1:** Characteristics of patients who were excluded from the analysis (adult patients without epinephrine administration).

Variables		South Korea		Singapore	
		N = 89,081		N = 5084	
		N	%	N	%
Age	median (IQR)	72 (58, 82)		72 (59, 84)	
Gender	male	56,187	63.1	3120	61.4%
	female	32,894	36.9	1964	38.6%
Year	2018	-	-	1450	28.5%
	2019	10,214	11.5	1659	32.6%
	2020	24,626	27.6	1975	38.8%
	2021	26,349	29.6	-	-
	2022	27,892	31.3	-	-
Witnessed arrest	yes	42,323	47.5	2463	48.4%
	no	46,758	52.5	2621	51.6%
Location of arrest	private	55,247	62.0	4346	85.5%
	public	33,834	38.0	738	14.5%
Bystander CPR	yes	43,944	49.3	2879	56.6%
	no	45,137	50.7	2205	43.4%
Initial shockable rhythm	yes	9669	10.9	730	14.4%
	no	79,412	89.1	4354	85.6%
Prehospital defibrillation	yes	13,389	15.0	898	17.7%
	no	75,692	85.0	4186	82.3%
PCI (yes)	yes	5027	5.6	229	5.1%
TTM (yes)	yes	3138	3.5	250	5.5%
ECMO (yes)	yes	897	1.0	10	0.2%
Any ROSC (yes)	yes	29,579	33.2	1396	30.9%
Survival to discharge	yes	7180	8.1	403	7.9%
Good neurologic recovery	CPC 1, 2	4769	5.4	322	6.3%
	CPC 3, 4, 5	84,312	94.6	4759	93.7%

**Table A2:** Scene time interval and field arrival to epinephrine time categorized by median value.

	Survival to Discharge					Good Neurologic Recovery					Total	
	No		Yes		*p*-Value	No		Yes		*p*-Value		
	N	%	N	%		N	%	N	%		N	%
South Korea	17,760	94.1	1107	5.9		18,341	97.2	526	2.8		18,867	
scene time interval and field arrival to epinephrine time categorized by median value												
short stay and early epinephrine	5621	92.4	460	7.6	<0.01	5852	96.2	229	3.8	<0.01	6081	32.2
short stay and late epinephrine	2588	94.9	140	5.1	<0.01	2666	97.7	62	2.3	<0.01	2728	14.5
long stay and early epinephrine	2644	93.4	186	6.6	0.14	2731	96.5	99	3.5	0.02	2830	15.0
long stay and late epinephrine	6907	95.6	321	4.4	<0.01	7092	98.1	136	1.9	<0.01	7228	38.3
scene time interval categorized by median value and field arrival to epinephrine time categorized by 10 min												
short stay and early epinephrine	4772	92.3	399	7.7	<0.01	4973	96.2	198	3.8	<0.01	5171	27.4
short stay and late epinephrine	3437	94.5	201	5.5		3545	97.4	93	2.6		3638	19.3
long stay and early epinephrine	1851	93.1	137	6.9		1919	96.5	69	3.5		1988	10.5
long stay and late epinephrine	7700	95.4	370	4.6		7904	97.9	166	2.1		8070	42.8
Singapore	4079	97.5	105	2.5		4131	98.7	53	1.3		4177	
scene time interval and field arrival to epinephrine time categorized by median value												
short stay and early epinephrine	1163	96.5	42	3.5	<0.001	1187	98.5	18	1.5	0.006	1205	28.8
short stay and late epinephrine	702	98.2	13	1.8		709	99.2	6	0.8		715	17.1
long stay and early epinephrine	725	96.2	29	3.8		736	97.6	18	2.4		754	18
long stay and late epinephrine	1489	98.6	21	1.4		1499	99.3	11	0.7		1510	36.1
scene time interval categorized by median value and field arrival to epinephrine time categorized by 10 min												
short stay and early epinephrine	466	95.7	21	4.3	<0.01	479	98.4	8	1.6	<0.01	487	11.6
short stay and late epinephrine	1399	97.6	34	2.4		1417	98.9	16	1.1		1433	34.2
long stay and early epinephrine	140	92.7	11	7.3		143	94.7	8	5.3		151	3.6
long stay and late epinephrine	2074	98.2	39	1.8		2092	99.0	21	1.0		2113	50.5

**Table A3:** Multivariable logistic regression of scene time interval and field arrival to epinephrine time for clinical outcome in OHCA using median value for field arrival to epinephrine administration time.

		Total	Outcome		Unadjusted			Adjusted *		
			N	%	OR	95% CI		AOR	95% CI	
South Korea										
Survival to discharge		18,867	1107	5.9						
Median STI and Median FEI	short stay early epinephrine	5621	460	7.6	1.76	1.52	2.04	2.12	1.80	2.50
	short stay late epinephrine	2588	140	5.1	1.16	0.95	1.43	1.06	0.85	1.33
	long stay early epinephrine	2644	186	6.6	1.51	1.26	1.82	1.72	1.40	2.12
	long stay late epinephrine	6907	321	4.4	reference			reference		
Good neurologic recovery		18,867	526	2.8						
Median STI and Median FEI	short stay early epinephrine	5852	229	3.8	2.04	1.65	2.53	2.49	1.95	3.20
	short stay late epinephrine	2666	62	2.3	1.21	0.90	1.64	1.20	0.85	1.69
	long stay early epinephrine	2731	99	3.5	1.89	1.45	2.46	2.18	1.62	2.94
	long stay late epinephrine	7092	136	1.9	reference			reference		
Singapore										
Survival to discharge		4184	105	2.5						
Median STI and Median FEI	short stay early epinephrine	1205	42	3.5	2.56	1.53	4.43	2.48	1.42	4.46
	short stay late epinephrine	715	13	1.8	1.31	0.64	2.61	0.78	0.37	1.6
	long stay early epinephrine	754	29	3.8	2.84	1.61	5.07	3.00	1.63	5.63
	long stay late epinephrine	1510	21	1.4	reference			reference		
Good neurologic recovery		4184	53	1.3						
Median STI and Median FEI	short stay early epinephrine	1205	18	1.5	2.07	0.99	4.53	1.73	0.77	4.07
	short stay late epinephrine	715	6	0.8	1.15	0.4	3.04	0.58	0.19	1.62
	long stay early epinephrine	754	18	2.4	3.33	1.59	7.32	2.89	1.26	6.89
	long stay late epinephrine	1510	11	0.7	reference			reference		

* Adjusted for age by 10 years, gender, witness, bystander CPR, initial shockable rhythm, prehospital advanced airway, prehospital defibrillation, number of prehospital epinephrine injections, response time, transport time, study period by year.

**Table A4:** Interaction analysis for scene time interval and epinephrine administration time with clinical outcome using median value for field arrival to epinephrine administration time.

Country	Outcome		Scene Time Interval Categorized by Median Value					
			Short Scene Time Interval			Long Scene Time Interval		
			AOR	95% CI		AOR	95% CI	
South Korea	Survival to discharge	early epinephrine by median value	2.00	1.60	2.49	1.72	1.40	2.12
		late epinephrine by median value	reference			reference		
	Good neurologic recovery	early epinephrine by median value	2.09	1.50	2.91	2.18	1.62	2.94
		late epinephrine by median value	reference			reference		
Singapore	Survival to discharge	early epinephrine by median value	3.37	1.73	6.94	2.92	1.54	5.62
		late epinephrine by median value	reference			reference		
	Good neurologic recovery	early epinephrine by median value	2.96	1.1	8.93	3.46	1.45	8.62
		late epinephrine by median value	reference			reference		
			Epinephrine categorized by median value					
			Early epinephrine			Late epinephrine		
South Korea	Survival to discharge	short scene time interval	1.23	1.01	1.50	1.06	0.85	1.33
		long scene time interval	reference			reference		
	Good neurologic recovery	short scene time interval	1.14	0.86	1.52	1.20	0.85	1.69
		long scene time interval	reference			reference		
Singapore	Survival to discharge	short scene time interval	0.90	0.53	1.56	0.77	0.35	1.65
		long scene time interval	reference			reference		
	Good neurologic recovery	short scene time interval	0.62	0.28	1.34	0.54	0.17	1.59
		long scene time interval	reference			reference		

Adjusted for age by 10 years, gender, witness, bystander CPR, initial shockable rhythm, prehospital advanced airway, prehospital defibrillation, number of prehospital epinephrine injections, response time, transport time, study period by year.
